# Circumferential Aneurysm Wall Enhancement Predicts Recanalization After Stent‐Assisted Coiling in Small Unruptured Intracranial Aneurysms

**DOI:** 10.1002/brb3.70898

**Published:** 2025-09-21

**Authors:** Qiu‐Yi Jiang, Xin‐Lei Ruan, Rui Chen, Zhong‐Song Shi

**Affiliations:** ^1^ Department of Neurosurgery Sun Yat‐Sen Memorial Hospital Sun Yat‐Sen University Guangzhou China; ^2^ RNA Biomedical Institute Sun Yat‐Sen Memorial Hospital Sun Yat‐Sen University Guangzhou China; ^3^ Guangdong Province Key Laboratory of Brain Function and Disease Sun Yat‐Sen University Guangzhou China

**Keywords:** aneurysm wall enhancement, endovascular treatment, recanalization, unruptured intracranial aneurysm, vessel wall imaging

## Abstract

**Background:**

Circumferential aneurysm wall enhancement (CAWE) on high‐resolution vessel wall imaging (HR‐VWI) as a vessel wall inflammation marker is associated with unruptured intracranial aneurysms (UIAs) instability and recanalization after endovascular treatment. This study evaluates the association of CAWE with recanalization of small UIAs (< 10 mm) treated with stent‐assisted coiling (SAC) or coiling alone and aims to develop a prediction model for recanalization based on CAWE.

**Methods:**

We analyzed patients with small saccular UIAs undergoing 3T HR‐VWI and treated with SAC or coiling alone between October 2018 and May 2024. A four‐grade scale assessed aneurysm wall enhancement (none, focal thick wall enhancement, thin CAWE, and thick CAWE). The aneurysm‐to‐pituitary stalk contrast ratio (CRstalk) quantified enhancement. We investigated the relationship between CAWE and recanalization and developed a recanalization prediction model.

**Results:**

Sixty‐five patients with 69 UIAs were included; 11 aneurysms (15.9%) exhibited CAWE, and 10 had a CRstalk ≥ 0.5. Sixty aneurysms (87%) received SAC. Recanalization occurred in 14 of 69 aneurysms (20.3%), assessed by DSA follow‐up at 12.6 months. Multivariate analysis revealed that aneurysm size, CAWE, and CRstalk ≥ 0.5 predicted recanalization undergoing coiling with or without stent assistance. A scoring prediction model was created using aneurysm size, treatment, embolization occlusion, and CAWE, with scores ranging from 0 to 6, where scores ≥ 3 indicated high risk and a C‐statistic of 0.892 demonstrated excellent discrimination.

**Conclusions:**

CAWE on HR‐VWI is a significant imaging marker for predicting recanalization in small UIAs undergoing SAC. The proposed recanalization risk scale needs validation in larger studies.

## Graphical abstract

Circumferential aneurysm wall enhancement (CAWE) on high‐resolution vessel wall imaging can predict recanalization in small unruptured intracranial aneurysms following coiling with or without stent assistance. A scoring‐based prediction model has been developed to evaluate the risk of recanalization, incorporating factors of aneurysm size, CAWE, endovascular treatment, and embolization occlusion grade.

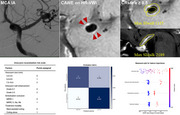

## 1 Introduction

The rupture of intracranial aneurysms leading to subarachnoid hemorrhage can be effectively addressed through endovascular treatment or surgical clipping. The International Subarachnoid Aneurysm Trial study indicates that over 80% of ruptured aneurysms are small sized (less than 10 mm) (Molyneux et al. 2002). Unruptured intracranial aneurysms (UIAs) of small size demonstrate a low risk of rupture and growth (Wiebers 2003; UCAS Japan Investigators et al. 2012; Greving et al. 2014; Backes et al. 2017). Notably, tiny UIAs (3 mm or smaller) are generally not recommended for preventative treatment (Malhotra et al. 2018). In the last 20 years, more intracranial aneurysms have been treated using safer endovascular techniques, and the size of treated UIAs has decreased over time (Salem et al. 2021; Khorasanizadeh et al. 2023). The modalities, including endovascular coiling, stent‐assisted coiling (SAC), balloon‐assisted coiling, flow diverters, and Woven EndoBridge devices, are effective treatment options for small and medium‐sized UIAs with wide necks (Boisseau et al. 2023; Pierot et al. 2019; Hanel et al. 2020, H. Zhang et al. 2023; Girot et al. 2021). The use of flow diverters for small UIAs carries a risk of 2% for major stroke or neurological death (Pierot et al. 2019; Hanel et al. 2020, H. Zhang et al. 2023). Coiling alone and coiling with stent or balloon assistance have limitations due to the associated rates of aneurysm recanalization ranging from 8% to 33% (Boisseau et al. 2023; Pierot et al. 2022; Duan et al. 2023). Several factors related to patients and aneurysm morphology are linked to recanalization after coiling, such as smoking, aneurysm size, neck size, and aneurysm location (Pierot et al. 2022; Duan et al. 2023; Futchko et al. 2018). The recanalization rates vary between 5% and 22% undergoing SAC, which lowers recanalization rates without increasing complication risks compared to using coiling alone (Boisseau et al. 2023; Pierot et al. 2022; Lim et al. 2021).

High‐resolution vessel wall imaging (HR‐VWI) has become an essential MRI technique for evaluating the thickness and enhancement of aneurysms (Sanchez et al. 2024). Aneurysm wall enhancement (AWE) is a crucial imaging biomarker that indicates the histological features of degenerative changes, neovascularization, and inflammation within the aneurysm wall (Shimonaga et al. 2018). Circumferential AWE (CAWE) and quantitative analysis with a higher contrast ratio measurement are more strongly associated with aneurysm instability, an important indicator for preventative treatments in UIAs to help reduce their significant risk of rupture (Edjlali et al. 2018; Omodaka et al. 2018; Wu et al. 2022; Veeturi et al. 2025). Preoperative AWE in saccular UIAs predicts clinical outcome at 6‐month follow‐up after surgical clipping (Hu et al. 2023). CAWE before endovascular treatment can predict aneurysm recanalization or enlargement of unruptured vertebrobasilar dissection aneurysms following SAC or stenting alone (Y. Zhang et al. 2018). In the case of saccular UIAs, CAWE before endovascular coiling is also associated with aneurysm recanalization (Hara et al. 2022). There is limited evidence regarding the prediction of pretreated AWE in recanalization following SAC in small UIAs (< 10 mm). This study aimed to investigate the association between CAWE and quantitatively measured aneurysm wall enhancement in 3T HR‐VWI with recanalization of small UIAs (< 10 mm) treated with coiling with or without stent assistance, mainly focusing on SAC, as well as to develop a prediction model for recanalization based on CAWE.

## 2 Methods

### 2.1 Study Population

We selected consecutive patients with UIA for initial preventative treatment using endovascular and surgical techniques from the HR‐VWI aneurysm database at our institution between October 2018 and May 2024. The study included adult patients (older than 18 years) with small saccular UIAs (ranging from 3 to 10 mm) treated with endovascular coiling alone or with SAC. We excluded patients with aneurysms in the extracranial or cavernous sinus sections of the internal carotid artery. All patients with aneurysms had an identified MR angiography and a favorable quality of HR‐VWI images for assessment. Furthermore, all participants received digital subtraction angiography follow‐up at least 3 months after endovascular treatment. The study received approval from the ethics committee at our institution.

We collected the data on demographics, laboratory examination, radiological, and clinical characteristics. The morphological factors of aneurysm included the maximum aneurysm size, neck size, location, size ratio, aspect ratio, dome‐to‐neck ratio, and bifurcation or sidewall pattern. We assessed the risk of aneurysm rupture and growth using the PHASES and ELAPSS scores.

### 2.2 Aneurysm Wall Enhancement on HR‐VWI

Patients underwent brain MR for HR‐VWI on a 3.0T MRI scanner (Achieva TX, Philips Healthcare, Best, the Netherlands) with a 32‐channel head coil. The HR‐VWI protocol with precontrast and postcontrast sequences was described in our previous study. The assessment of AWE in the pretreatment MR was conducted using both a subjective AWE scale and an objective measurement of the signal intensity of the aneurysm wall, as described in earlier studies (Edjlali et al. 2018; Omodaka et al. 2018; Wu et al. 2022; Roa et al. 2020). A four‐grade scale was used to evaluate AWE. Grade 0 indicates no definite or questionable enhancement compared with the precontrast sequence. Grade 1 indicates focal thick enhancement with thickness > 1 mm. Grade 2 indicates thin, circumferential enhancement of the entire wall with thickness ≤ 1 mm. Grade 3 indicates thick, circumferential enhancement of the entire wall with thickness > 1 mm, or thin circumferential enhancement of the entire wall with focal enhancement thickness > 1 mm. AWE grades 2 and 3 were classified as CAWE (Edjlali et al. 2018). For quantitative analysis, the maximum signal intensity of the aneurysmal wall (SIwall) and the pituitary stalk (SIstalk) was measured. The aneurysm‐to‐pituitary stalk contrast ratio (CRstalk) was then calculated to reflect the degree of AWE, using the following formula: CRstalk = SIwall/SIstalk (Omodaka et al. 2018; Wu et al. 2022; Roa et al. 2020).

### 2.3 Endovascular Treatment and Angiographic Follow‐Up

Patients underwent endovascular treatment using coiling, with or without stent placement, while under general anesthesia. SAC was chosen when an aneurysm had a wide neck (≥4 mm) or a dome‐to‐neck ratio < 2. Bare platinum coils and Enterprise‐2 stents (Codman Neuro) were utilized in this study. Patients who received SAC were prescribed dual antiplatelet regimens, including Aspirin, clopidogrel, and platelet inhibition testing.

The modified Raymond‐Roy classification (MRRC) was utilized to evaluate the degree of aneurysm embolization, assigning grades I, II, and III (with subcategories IIIa and IIIb) (Mascitelli et al. 2015). MRRC grade I indicates complete occlusion, while grades I and II are considered satisfactory occlusion based on the immediate post‐embolization angiogram. The modified Rankin Scale was assessed at discharge, outpatient visits, and inpatient clinics. Follow‐up digital subtraction angiography was conducted at 3–6 months, 12 months, and 24 months posttreatment. Evaluations focused on coil compaction in the sac and neck and aneurysm regrowth. An increase in the MRRC grade on follow‐up angiography compared to immediate posttreatment angiograms is considered an aneurysm recanalization (Pierot et al. 2022; Duan et al. 2023).

### 2.4 Data Analysis

R version 4.2.2 was used for statistical analysis. Two researchers, blinded to the clinical and radiological data, independently assessed the AWE and MRRC pattern. The inter‐rater agreement was measured using the Cohen *κ* coefficient and intra‐class correlation coefficient. A third researcher resolved the disagreement. We analyzed the continuous variables using Student's *t*‐test or Mann‐Whitney *U* test, and categorical variables by chi‐square or Fisher's exact test. Then, we performed the univariate and multivariate logistic regression analyses to determine the independent factors related to aneurysm recanalization. The absence of multicollinearity was evaluated using the variance inflation factor (VIF) < 2.5; VIFs were between 1.05 and 1.78 in our models, suggesting the absence of multicollinearity. A *p*‐value < 0.05 was considered statistically significant.

By integrating the findings from this study with previous research and clinical practice experience, we identified predictive factors to construct a multivariable logistic regression model. Following the established method, we converted aneurysm size into a categorical variable to create a clinically applicable scoring system, assigning a value of 1 to the minimum nonzero regression coefficient. Next, we calculated the ratio of each predictor's regression coefficient to this baseline value, rounded it to the nearest integer, and assigned values accordingly to develop an aneurysm recanalization risk score. We meticulously evaluated the performance of this model using receiver operating characteristic (ROC) curve analysis, a confusion matrix, and a calibration curve. The critical threshold for risk stratification was determined based on the optimal cutoff value identified from the ROC curve. We further elucidated the multivariable logistic regression model using the SHapley Additive exPlanations (SHAP) method to enhance the interpretability of the model (Sullivan et al. 2004).

## 3 Results

### 3.1 Patient, Aneurysm, and HR‐VWI Characteristics

Two hundred eight consecutive patients with UIAs receiving pretreatment HR‐VWI assessment and endovascular treatment were identified during the study period. Sixty‐five patients with 69 saccular UIAs were included in this study according to the predefined eligibility criteria. The mean age was 57.3 ± 10.8 years, and 40 (58%) were female. The mean size of aneurysms was 5.7 ± 1.8 mm, with 25 (36%) presenting with irregular shapes. All aneurysms had a maximum size of less than 10 mm, with 3.0–4.9 mm size in 26 aneurysms, 5.0–6.9 mm in 29, and 7.0–9.9 mm in 14. Twenty‐five aneurysms had a wide‐neck (≥ 4.0 mm), and 65 aneurysms had a dome‐to‐neck ratio < 2. Forty‐six (66.7%) aneurysms were located in the internal carotid artery, 11 (15.9%) aneurysms were in the middle cerebral artery, five (7.2%) aneurysms were in the anterior communicating artery, four (5.8%) in the posterior communicating artery, and three (4.3%) in the posterior circulation. The PHASES score ranged from 0 to 8, and the ELAPSS score ranged from 5 to 23.

A four‐grade scale AWE at 3T HR‐VWI showed 47 as grade 0, 11 as grade 1, 4 as grade 2, and 7 as grade 3. The pattern of CAWE included AWE grades 2 and 3, which were observed in 11 aneurysms (15.9%). The interreader agreement for the identification of AWE was excellent, with *κ* = 0.90. The mean value of CRstalk was 0.33 ± 0.16 in this study. The CRstalk value ≥ 0.5 related to aneurysm instability in saccular UIAs was previously shown. Ten of 69 aneurysms (14.5%) had a CRstalk ≥ 0.5. The interreader agreement for the measurement of CRstalk was excellent, with intra‐class correlation coefficient = 0.99. The characteristics of the patient, aneurysm, and image are shown in Table 1.

**TABLE 1:** Characteristics of unruptured intracranial aneurysms.

	Total (*n* = 69)	Recanalization (*n* = 14)	No recanalization (*n* = 55)	*p* value
Age (year)	57.3 ± 10.8	57.1 ± 12.5	57.4 ± 10.5	0.946
Female	40 (58.0%)	4 (28.6%)	36 (65.5%)	0.017
Male	29 (42.0%)	10 (71.4%)	19 (34.5%)	0.017
Hypertension	32 (46.4%)	6 (42.9%)	26 (47.3%)	1.000
Diabetes	10 (14.5%)	2 (14.3%)	8 (14.5%)	1.000
Dyslipidemia	5 (7.2%)	2 (14.3%)	3 (5.5%)	0.266
Smoking	13 (18.8%)	6 (42.9%)	7 (12.7%)	0.028
Aneurysm size (mm)	5.7 ± 1.8	7.5 ± 2.0	5.2 ± 1.5	< 0.001
3.0–4.9 mm	26 (37.7%)	2 (14.3%)	24 (43.6%)	
5.0–6.9 mm	29 (42.0%)	3 (21.4%)	26 (47.3%)	
7.0–9.9 mm	14 (20.3%)	9 (64.3%)	5 (9.1%)	
Neck size ≥4.0 mm	25 (36.2%)	8 (57.1%)	17 (30.9%)	0.131
Site of aneurysm				0.022
ICA	46 (66.7%)	5 (35.7%)	41 (74.5%)	
MCA	11 (15.9%)	4 (28.6%)	7 (12.7%)	
AComA	5 (7.2%)	2 (14.3%)	3 (5.5%)	
PComA	4 (5.8%)	1 (7.1%)	3 (5.5%)	
PC	3 (4.3%)	2 (14.3%)	1 (1.8%)	
Bifurcation aneurysm	22 (31.9%)	8 (57.1%)	14 (25.5%)	0.051
PHASES score	1.0 (0.0–4.0)	5.0 (3.0–6.0)	1.0 (0.0–3.0)	< 0.001
ELAPSS score	12.0 (9.0–15.0)	15.5 (14.0–19.0)	11.0 (7.5–15.0)	0.001
AWE				< 0.001
Grade 0	47 (68.1%)	3 (21.4%)	44 (80.0%)	
Grade 1	11 (15.9%)	3 (21.4%)	8 (14.5%)	
Grade 2	4 (5.8%)	2 (14.3%)	2 (3.6%)	
Grade 3	7 (10.1%)	6 (42.9%)	1 (1.8%)	
CAWE, grade 2 and 3	11 (15.9%)	8 (57.1%)	3 (5.5%)	< 0.001
CR stalk	0.33 ± 0.16	0.50 ± 0.16	0.28 ± 0.12	< 0.001
Endovascular treatment				
Stent‐assisted coiling	60 (87.0%)	9 (64.3%)	51 (92.7%)	0.014
Coiling alone	9 (13.0%)	5 (35.7%)	4 (7.3%)	0.014
Embolization occlusion				0.032
MRRC grade I	48 (69.6%)	7 (50.0%)	41 (74.5%)	
MRRC grade II	16 (23.2%)	7 (50.0%)	9 (16.4%)	
MRRC grade IIIa+IIIb	5 (7.2%)	0 (0.0%)	5 (9.1%)	
Coil compaction	13 (18.8%)	11 (78.6%)	2 (3.6%)	< 0.001

Abbreviations: AComA, anterior communicating artery; AWE, aneurysm wall enhancement; CAWE, circumferential aneurysm wall enhancement; CR, contrast ratio; ICA, internal carotid artery; MCA, middle cerebral artery; MRRC, modified Raymond‐Roy classification; PC, posterior circulation; PComA, posterior communicating artery.

### 3.2 Aneurysm Recanalization After Endovascular Treatment

**FIGURE 1:**
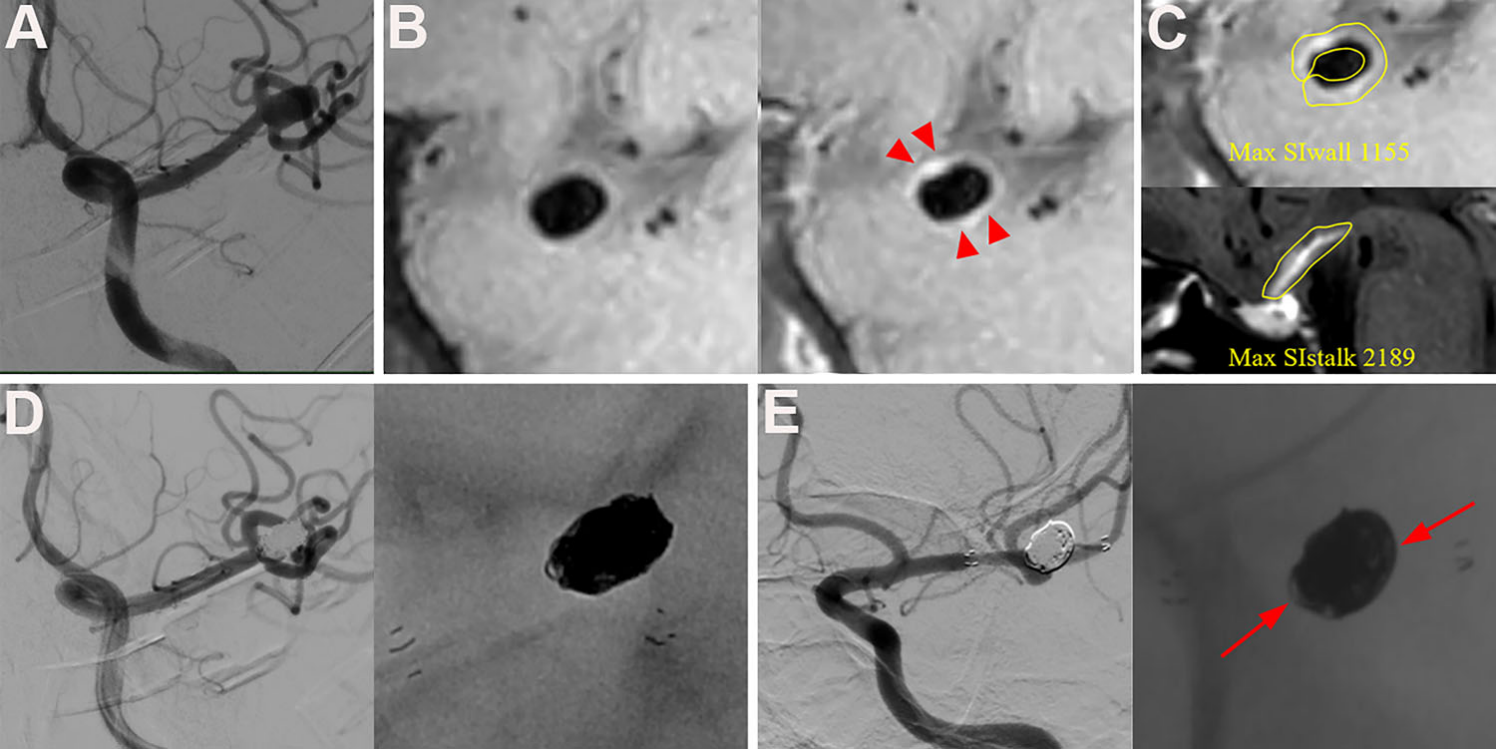
An unruptured 8.8‐mm left middle cerebral artery bifurcation saccular aneurysm shown on digital subtraction angiography (A) was treated by stent‐assisted coiling. The pretreatment HR‐VWI showed CAWE on the precontrast and postcontrast sequences (red arrowheads) (B) and the quantitative CRstalk value as 0.53 (C). The aneurysm received satisfactory occlusion by stent‐assisted coiling, shown on the immediate post‐embolization angiogram (D). A follow‐up digital subtraction angiography at 9 months after treatment showed minor recanalization with coil compaction in the aneurysmal neck and sac (red arrow) (E).

Sixty aneurysms received SAC, and nine aneurysms were treated by coiling only. Complete occlusion was shown as MRRC grade I in 48 aneurysms at the immediate embolization angiogram. The satisfactory embolization occlusion was achieved in 64 aneurysms, with 48 in MRRC grade I and 16 in MRRC grade II. There were no ischemic or hemorrhagic complications related to endovascular treatment. Aneurysm recanalization was detected in 14 of 69 (20.3%), as shown by digital subtraction angiography follow‐up at 12.6 (3–23) months. Of these 14 recanalized aneurysms, 13 exhibited coil compaction (Figure 1). All 65 patients had a favorable clinical outcome with an mRS scale of less than 1 at 90 days and are monitored regularly.

### 3.3 CAWE and CRstalk As Predictors of Aneurysm Recanalization

In the univariable analysis showed in Table 1, recanalized aneurysms were more prevalent in male patients (71.4% vs. 34.5%, *p* = 0.017) and smokers (42.9% vs. 12.7%, *p* = 0.028). Recanalized aneurysms were significantly larger (7.5 mm vs. 5.2 mm, *p *< 0.001) and were less frequently located in the internal carotid artery (35.7% vs. 74.5%, *p* = 0.007). Higher PHASES and ELAPSS scores were observed in the recanalized aneurysms. The AWE pattern was more pronounced in recanalized aneurysms compared to non‐recanalized ones (all AWE, 78.6% vs. 20.0%, *p *< 0.001; all CAWE, 57.1% vs. 5.5%, *p *< 0.001; CAWE grade 3, 42.9% vs. 1.8%, *p *< 0.001). A higher mean CRstalk value (0.50 vs. 0.28, *p *< 0.001) and higher percent of CRstalk ≥ 0.5 (50.0% vs. 5.5%, *p *< 0.001) were exhibited in the recanalized aneurysms. Recanalized aneurysms were more frequently treated with coiling alone (35.7% vs. 7.3%, *p* = 0.014). Coil compaction occurred significantly more often in recanalized aneurysms (78.6% vs. 3.6%, *p *< 0.001).

In the multivariate analysis, after adjusting variables of smoking, aneurysm size, location in the internal carotid artery, complete occlusion at immediate embolization, coiling alone, and CAWE with grades 2 and 3, the results showed that aneurysm size (OR 2.19, 95% CI 1.28–4.53, *p* = 0.012) and CAWE with grades 2 and 3 (OR 28.32, 95% CI 3.87–364.92, *p* = 0.003) were independently associated with recanalization after endovascular treatment. In the multivariate analysis, after adjusting variables of smoking, aneurysm size, location in the internal carotid artery, complete occlusion at immediate embolization, coiling alone, and CRstalk ≥ 0.5, the results showed that aneurysm size (OR 2.19, 95% CI 1.27–4.53, *p* = 0.013) and CRstalk ≥ 0.5 (OR 11.09, 95% CI 1.51–116.15, *p* = 0.024) were independently associated with recanalization after endovascular treatment (Table 2). In the univariable analysis of 60 aneurysms treated with SAC, the above factors were also related to aneurysm recanalization (Table S1). Aneurysm size, CAWE with grades 2 and 3, and CRstalk ≥ 0.5 remained independent risk factors for recanalization (Table 3).

**TABLE 2:** Multivariable logistic regression analysis of risk factors associated with aneurysm recanalization after stent‐assisted coiling and coiling alone.

	Model 1		Model 2	
Variable	Odds ratio (95% CI)	*p* value	Odds ratio (95% CI)	*p* value
Aneurysm size	2.19 (1.28–4.53)	0.012	2.19 (1.27–4.53)	0.013
CAWE, grades 2 and 3	28.32 (3.87–364.92)	0.003	—	—
CR stalk ≥ 0.5	—	—	11.09 (1.51–116.15)	0.024

*Note*: Model 1 was adjusted for smoking, aneurysm size, aneurysm location in the internal carotid artery, aneurysm complete occlusion at immediate embolization, coiling alone, CAWE with grades 2 and 3, and Model 2 was adjusted for smoking, aneurysm size, aneurysm location in the internal carotid artery, aneurysm complete occlusion at immediate embolization, coiling alone, and CR stalk ≥ 0.5.

Abbreviations: CAWE, circumferential aneurysm wall enhancement; CI, confidence interval; CR, contrast ratio.

**TABLE 3:** Multivariable logistic regression analysis of risk factors associated with aneurysm recanalization after the subgroup of stent‐assisted coiling.

	Model 1		Model 2	
Variable	Odds ratio (95% CI)	*p* value	Odds ratio (95% CI)	*p* value
Aneurysm size	2.11 (1.18–4.77)	0.027	1.85 (1.12–3.39)	0.025
CAWE, grades 2 and 3	55.48 (4.72–1425.64)	0.004	—	—
CR stalk ≥ 0.5	—	—	14.24 (1.25–244.63)	0.040

*Note*: Model 1 was adjusted for smoking, aneurysm size, aneurysm location in the internal carotid artery, aneurysm complete occlusion at immediate embolization, CAWE with grades 2 and 3, and Model 2 was adjusted for smoking, aneurysm size, internal carotid artery, complete occlusion, and CR stalk ≥ 0.5.

Abbreviations: CAWE, circumferential aneurysm wall enhancement; CI, confidence interval; CR, contrast ratio.

### 3.4 Recanalization Risk Score Model

Based on the multivariable analyses, a recanalization risk scoring system integrating aneurysm size, CAWE, embolization occlusion, and treatment modality was developed for small UIAs less than 10 mm following SAC and coiling based on the multivariable analyses (Table 4). We selected CAWE grades 2 and 3 over the objective quantification of CRstalk in the risk score model for broad application across different medical providers, as the subjective evaluation of vessel wall enhancement requires less training and resources. The total score was 0–6 points, with scores ≥ 3 indicating a high risk of recanalization. The recanalization rates at each score level were as follows: 0, 6.3%; 1, 0.0%; 2, 20.0%; 3, 66.7%; 4, 60.0%; and 5, 100%. The median risk score was 1.0, and the mean score was 1.60 ± 1.46.

**TABLE 4:** Aneurysm recanalization risk scale.

Factors	Points assigned
Aneurysm size (mm)	
3.0–4.9	0
5.0–6.9	1
7.0–10.0	2
Aneurysm wall enhancement	
Grade 0–1	0
Grade 2–3	2
Embolization occlusion	
MRRC I	0
MRRC II, IIIa, IIIb	1
Treatment modality	
Stent‐assisted coiling	0
Coiling alone	1

**FIGURE 2:**
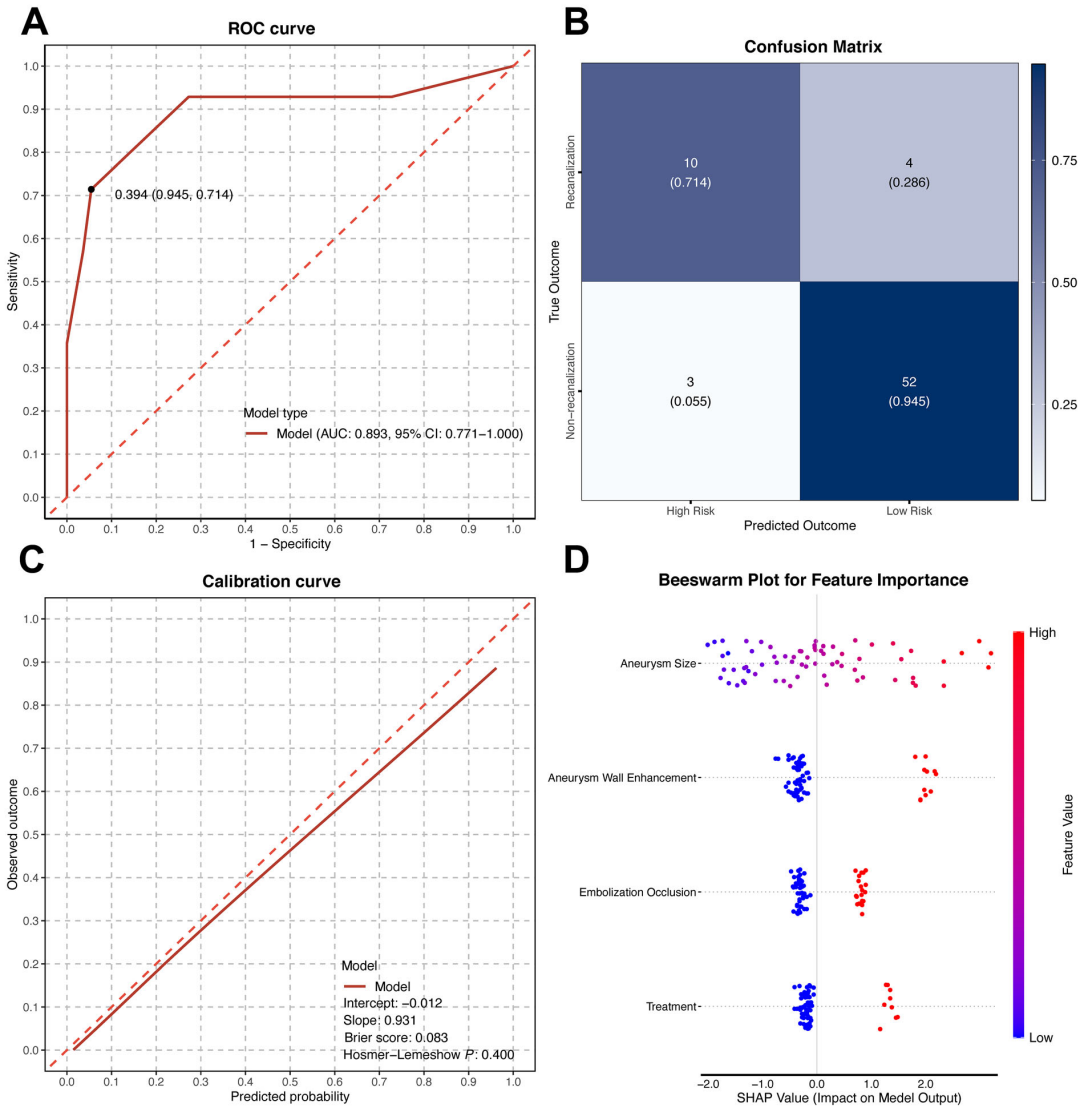
Performance evaluation and interpretation of the prediction model for aneurysm recanalization after coiling alone or stent‐assisted coiling in small unruptured intracranial aneurysms. (A) Receiver operating characteristic (ROC) curve demonstrating the model's discriminative ability. (B) Confusion matrix illustrating the classification performance of the model in predicting recanalization. (C) Calibration curve assessing agreement between predicted probabilities and observed outcomes. (D) Beeswarm plot of SHAP values depicting the relative contribution and directionality of each feature to the model prediction, with aneurysm size, aneurysm wall enhancement, embolization occlusion, and treatment modality ranked by importance. Each dot represents an individual sample, with color indicating the feature value (red: high, blue: low) and position on the *x*‐axis representing the SHAP value.

The risk scoring system demonstrated good discriminative ability and adequate calibration, indicating reasonable predictive performance. An area under the curve from ROC analysis was 0.893, with an optimal probability threshold of 0.394 determined by the Youden index (Figure 2A). The model of the confusion matrix achieved a sensitivity of 71.4%, specificity of 94.5%, accuracy of 89.9%, precision of 76.9%, and an F1 score of 74.0% (Figure 2B). Calibration analysis indicated strong concordance between predicted and observed outcomes with the *p*‐value of Hosmer–Lemeshow equal to 0.400 and the Brier score of 0.083 (Figure 2C). Feature importance and the direction of feature effects were visualized using a SHAP beeswarm plot. Ranking of features by their mean absolute SHAP values confirmed aneurysm size as the most influential predictor of recanalization, followed by CAWE, embolization occlusion, and treatment modality (Figure 2D).

## 4 Discussion

Our study found several factors associated with recanalization following SAC and coiling alone for small saccular UIAs less than 10 mm in size with wide necks. These factors include sex, smoking, aneurysm size, location, PHASES score, ELAPSS score, AWE pattern, CRstalk, type of endovascular treatment, embolization occlusion grade, and coil compaction. In our multivariate logistic regression analysis, after adjusting variables such as smoking, aneurysm size, location in the internal carotid artery, complete occlusion at immediate embolization, coiling alone, CAWE with grades 2 and 3, and CRstalk ≥ 0.5, we found that aneurysm size, CAWE with grades 2 and 3, and CRstalk ≥ 0.5 were independently associated with recanalization. Additionally, we developed a scoring‐based prediction model to assess the risk of recanalization using aneurysm size, type of endovascular treatment, embolization occlusion grade, and CAWE. The total score of this predictive model ranges from 0 to 6 points, with scores ≥ 3 indicating a high risk of recanalization. The model demonstrated excellent discriminative ability, achieving a C‐statistic of 0.892.

Coiling compaction and aneurysm growth were the main imaging findings for recanalization after coiling embolization (Pierot et al. 2022; Duan et al. 2023). In our study, over 85% of aneurysms were treated by SAC. Coiling compaction detected in the angiographic follow‐up was associated with aneurysm recanalization following SAC, even in aneurysms with initial complete embolization. Previous studies of histopathological examinations of recanalized aneurysms showed fresh thrombus, granulation tissue, and scar tissue in specific areas. Inadequate thrombus fibrosis with insufficient neck endothelialization and impaired smooth muscle cells with inflammatory cells infiltrating the aneurysm wall contribute to recanalization (Wang et al. 2022).

The imaging biomarker CAWE histologically reflects the entire aneurysm wall with inflammation (Sanchez et al. 2024; Shimonaga et al. 2018). Our study provides new evidence that pretreatment CAWE can predict aneurysm recanalization following SAC in small‐sized saccular UIAs less than 10 mm with wide necks. A recent study reported that CAWE and volume embolization ratio were associated with recanalization after endovascular treatment for saccular UIAs, in which two‐thirds of UIAs received coiling alone. Aneurysm recanalization was observed in 18 out of 67 aneurysms by follow‐up with magnetic resonance angiography. Of these recanalized aneurysms, 12 exhibited coil compaction, while six experienced growth (Hara et al. 2022). These findings support the potential of pretreatment CAWE as a predictive tool for aneurysm recanalization following SAC or coiling alone.

Enhancement of the aneurysm wall, aneurysm cavity, or stented parent artery wall from the follow‐up HR‐VWI was also found in aneurysms after endovascular treatment with coiling or flow diverters. These observations, which suggest either aneurysm healing or inflammatory reactions post‐endovascular coiling, remain debatable (Leber et al. 2024; Ladenhauf et al. 2024; Raz et al. 2022; Matsukawa et al. 2024; Quan et al. 2024). Recent studies have indicated a correlation between posttreatment AWE and aneurysm recanalization, particularly noting the largest recanalization diameter after initial complete occlusion through coiling, with a mean follow‐up period of 43 months (Leber et al. 2024). The relationship between posttreatment AWE and recanalization is notably more pronounced in coiled aneurysms with a maximum diameter exceeding 7.5 mm (Ladenhauf et al. 2024). The potential of posttreatment AWE as an indicator of aneurysm instability in coiled aneurysms that have achieved complete occlusion necessitates further investigation. HR‐VWI with contrast enhancement recently exited its effectiveness as an invasive tool for assessing aneurysm occlusion and in‐stent stenosis after flow diverter treatment (Raz et al. 2022; Matsukawa et al. 2024). Additionally, at the 1‐year follow‐up, AWE was observed in nearly 80% of complete occluded aneurysms and 100% of non‐occluded aneurysms. Stented parent artery wall enhancement was more announced in aneurysms with AWE (Raz et al. 2022). The occurrence of CAWE was noted in 23.8% of aneurysms before treatment, rising to 38% after flow diverter treatment, with an average follow‐up period of 8 months. Posttreatment AWE patterns observed at follow‐up did not significantly correlate with pretreatment AWE patterns and aneurysm occlusion in cases treated with flow diverters (Quan et al. 2024).

Objective quantification of the signal intensity of the aneurysm wall can provide an accurate assessment of AWE and reduce inconsistencies associated with subjective evaluations (Sanchez et al. 2024; Roa et al. 2020; Dier et al. 2025). The CRstalk measurement, which utilizes the maximal signal intensity, is a significant indicator of aneurysm instability (Roa et al. 2020). The value of CRstalk in unstable UIAs was higher than that of stable UIAs (Omodaka et al. 2018). In our previous study involving 75% saccular aneurysms smaller than 7 mm, a CRstalk value ≥ 0.5 was linked to aneurysm instability in UIAs (Wu et al. 2022). Besides using the subjective AWE scale as in previous studies, we discovered that the CRstalk value in recanalized aneurysms is higher than in non‐recanalized aneurysms. A CRstalk value of 0.5 or greater has been identified as a predictor of aneurysm recanalization after SAC. Recent advancements in three‐dimensional quantification mapping and histogram analysis have significantly improved the accuracy of evaluating aneurysm walls (Dier et al. 2025). A radiomics composite score provides a comprehensive assessment of the risk of UIA instability (Veeturi et al. 2025).

More small UIAs have been effectively managed in recent decades using surgical clipping and endovascular techniques. A scoring system has been recommended to improve treatment decision‐making for small UIAs measuring less than 7 mm. This system considers factors such as size, multiple aneurysms, anatomical location, family history of aneurysms, age, smoking history, and aneurysm shape. A retrospective review of the dataset revealed that nearly two‐thirds of the treated aneurysms had a score of more than 2, while three‐fourths of the non‐treated aneurysms under 7 mm had a score of 2 or lower (Salih et al. 2025). The aneurysm recanalization stratification scale was developed based on specific factors related to aneurysms and treatment methods. Aneurysm‐specific factors included aneurysm size at the cutoff of 10 mm, rupture, and presence of thrombus. Treatment‐related factors included stent assistance, flow diversion, and embolization occlusion scale. This score is more effective than Raymond Roy's occlusion classification for predicting the need for retreatment after endovascular therapy. However, this score does not apply to small UIAs less than 10 mm and does not consider the aneurysm vessel wall information (Ogilvy et al. 2015).

Our study introduced a scoring prediction model for recanalization after coiling with or without stent assistance, which incorporated aneurysm‐specific factors, treatment‐related factors, and the recently approved imaging technique for the aneurysm wall. The SHAP analysis clarified the degree and direction of influence for each predictor in the model. This approach revealed a hierarchical pattern of influence, with aneurysm size and CAWE as the most influential predictors, followed by embolization occlusion grade and treatment modality. CAWE reflects the active inflammatory remodeling and structural weakening of the aneurysm wall, both of which may weaken thrombus maturation and facilitate recanalization through new vascular channels. Larger aneurysms are more prone to mechanical instability and coil compression due to their association with unfavorable hemodynamic environments and lower coil filling density. This distribution of predictive influence underscores the multifactorial pathophysiology of recanalization and further provides quantitative evidence for the relative contributions of individual predictors. Although this new scale shows excellent discrimination for small UIAs less than 10 mm, it requires external validation in more extensive studies.

This study has some limitations. First, the subjects were drawn from a single center, and the sample size was relatively small, which could introduce selection bias. Additionally, the association of AWE with recanalization during long‐term follow‐up remains unclear, as our study had an average angiographic follow‐up period of only 12 months. Furthermore, the relationship between pretreatment AWE and aneurysm recanalization resulting from increased inflammatory responses could be insufficiently explored without histological examination.

## 5 Conclusions

Subjective assessment using CAWE and objective quantification with CRstalk on HR‐VWI serve as valuable markers for aneurysm vessel wall inflammation and can predict recanalization in small UIAs following SAC. A scoring‐based prediction model has been developed to evaluate the risk of recanalization, incorporating factors of aneurysm size, CAWE, type of endovascular treatment, and embolization occlusion grade. The proposed recanalization risk scale requires further investigation with a larger sample size.

## Author Contributions

Z.‐S.S. participated in the design of the present study, wrote the initial manuscript, and revised the manuscript. Q.‐Y.J. and X.‐L.R. wrote the initial manuscript. All authors participated in the interpretation and collection of the data, critically reviewed and edited the manuscript, and approved the final version.

## Ethics Statement

The study was reviewed and approved by the Ethics Committee of Sun Yat‐Sen Memorial Hospital, Sun Yat‐Sen University.

## Conflicts of Interest

The authors declare no conflicts of interest.

## Peer Review

The peer review history for this article is available at https://publons.com/publon/10.1002/brb3.70898.

## Supporting information




**Supplementary Material**: brb370898‐sup‐0001‐TableS1.docx

## Data Availability

The data that support the findings of this study are available from the corresponding author upon reasonable request.
