# Supplementary material for: Circumferential Aneurysm Wall Enhancement Predicts Recanalization After Stent‐Assisted Coiling in Small Unruptured Intracranial Aneurysms
Source: Brain Behav. 2025 Sep 21;15(9):e70898. doi: 10.1002/brb3.70898 (PMC12451054; doi:10.1002/brb3.70898)
Supplement: Supplementary file 1 — Supplementary Material: brb370898‐sup‐0001‐TableS1.docx [file BRB3-15-e70898-s001.docx]

| **Supplementary Table S1.** Characteristics of unruptured intracranial aneurysms with stent-assisted coiling treatment. | | | | |
| --- | --- | --- | --- | --- |
|  | **Total (n=60)** | **Recanalization (n=9)** | **No Recanalization (n=51)** | **P Value** |
| Age (yr) | 56.8 ± 11.1 | 56.7 ± 14.1 | 56.9 ± 10.6 | 0.961 |
| Female | 36 (60.0%) | 3 (33.3%) | 33 (64.7%) | 0.137 |
| Male | 24 (40.0%) | 6 (66.7%) | 18 (35.3%) | 0.137 |
| Hypertension | 27 (45.0%) | 3 (33.3%) | 24 (47.1%) | 0.495 |
| Diabetes | 10 (16.7%) | 2 (22.2%) | 8 (15.7%) | 0.637 |
| Dyslipidemia | 5 (8.3%) | 2 (22.2%) | 3 (5.9%) | 0.158 |
| Smoking | 10 (16.7%) | 4 (44.4%) | 6 (11.8%) | 0.034 |
| Aneurysm size (mm) | 5.5 ± 1.8 | 7.5 ± 2.3 | 5.2 ± 1.4 | <0.001 |
| 3.0-4.9 mm | 24 (40.0%) | 2 (22.2%) | 22 (43.1%) |  |
| 5.0-6.9 mm | 26 (43.3%) | 1 (11.1%) | 25 (49.0%) |  |
| 7.0-9.9 mm | 10 (16.7%) | 6 (66.7%) | 4 (7.8%) |  |
| Neck size ≥4.0 mm | 24 (40.0%) | 7 (77.8%) | 17 (33.3%) | 0.023 |
| Site of aneurysm |  |  |  | 0.010 |
| ICA | 42 (70.0%) | 3 (33.3%) | 39 (76.5%) |  |
| MCA | 10 (16.7%) | 4 (44.4%) | 6 (11.8%) |  |
| AComA | 3 (5.0%) | 0 (0.0%) | 3 (5.9%) |  |
| PComA | 2 (3.3%) | 0 (0.0%) | 2 (3.9%) |  |
| PC | 3 (5.0%) | 2 (22.2%) | 1 (2.0%) |  |
| Bifurcation aneurysm | 16 (26.7%) | 4 (44.4%) | 12 (23.5%) | 0.230 |
| PHASES score | 1.0 (0.0-4.0) | 5.0 (3.0-6.0) | 1.0 (0.0-3.0) | 0.001 |
| ELAPSS score | 11.5 (8.8-15.0) | 16.0 (14.0-21.0) | 11.0 (7.0-15.0) | 0.003 |
| AWE |  |  |  | <0.001 |
| Grade 0 | 44 (73.3%) | 2 (22.2%) | 42 (82.4%) |  |
| Grade 1 | 9 (15.0%) | 2 (22.2%) | 7 (13.7%) |  |
| Grade 2 | 3 (5.0%) | 2 (22.2%) | 1 (2.0%) |  |
| Grade 3 | 4 (6.7%) | 3 (33.3%) | 1 (2.0%) |  |
| CAWE, grade 2 and 3 | 7 (11.7%) | 5 (55.6%) | 2 (3.9%) | <0.001 |
| CR stalk | 0.32 ± 0.15 | 0.51 ± 0.17 | 0.28 ± 0.12 | <0.001 |
| Embolization occlusion |  |  |  | 0.053 |
| MRRC grade I | 41 (68.3%) | 4 (44.4%) | 37 (72.5%) |  |
| MRRC grade II | 14 (23.3%) | 5 (55.6%) | 9 (17.6%) |  |
| MRRC grade IIIa+IIIb | 5 (8.3%) | 0 (0.0%) | 5 (9.8%) |  |

AComA, indicates anterior communicating artery; AWE, aneurysm wall enhancement; CAWE, circumferential aneurysm wall enhancement; CR, contrast ratio; ICA, internal carotid artery; MCA, middle cerebral artery; MRRC, Modified Raymond-Roy Classification; PComA, posterior communicating artery; PC, posterior circulation.
